# *In Vivo* Immunomodulation and Lipid Peroxidation Activities Contributed to Chemoprevention Effects of Fermented Mung Bean against Breast Cancer

**DOI:** 10.1155/2013/708464

**Published:** 2013-04-22

**Authors:** Swee Keong Yeap, Hamidah Mohd Yusof, Nurul Elyani Mohamad, Boon Kee Beh, Wan Yong Ho, Norlaily Mohd Ali, Noorjahan Banu Alitheen, Soo Peng Koh, Kamariah Long

**Affiliations:** ^1^Institute of Bioscience, Universiti Putra Malaysia, Serdang, Selangor, Malaysia; ^2^Department of Cell and Molecular Biology, Faculty of Biotechnology and Biomolecular Science, Universiti Putra Malaysia, 43400 Serdang, Selangor, Malaysia; ^3^Department of Bioprocess Technology, Faculty of Biotechnology and Biomolecular Science, Universiti Putra Malaysia, 43400 Serdang, Selangor, Malaysia; ^4^Biotechnology Research Centre, Malaysian Agricultural Research and Development Institute (MARDI), 43400 Serdang, Selangor, Malaysia

## Abstract

Mung bean has been reported to have antioxidant, cytotoxic, and immunomodulatory effects *in vitro*. Fermented products are reported to have enhanced immunomodulation and cancer chemopreventive effects. In this study, fermented mung bean treatments *in vivo* were studied by monitoring tumor development, spleen immunity, serum cytokine (interleukin 2 and interferon gamma) levels, and spleen/tumor antioxidant levels after injection with low and high risk 4T1 breast cancer cells. Pretreatment with fermented mung bean was associated with delayed tumor formation in low risk mice. Furthermore, this treatment was connected with higher serum anticancer cytokine levels, spleen T cell populations, splenocyte cytotoxicity, and spleen/tumor antioxidant levels. Histopathological evaluation of fermented mung bean treated tumor revealed lower event of mitotic division. On the other hand, antioxidant and nitric oxide levels that were significantly increased in the untreated mice were inhibited in the fermented mung bean treated groups. These results suggested that fermented mung bean has potential cancer chemoprevention effects through the stimulation of immunity, lipid peroxidation, and anti-inflammation.

## 1. Introduction

Breast cancer is the most common type of cancer and has higher incidence in industrialized countries like Europe and United State than in developing countries such as China and India. This phenomenon has been related to different life style especially to the Asian diet and their food preparation methods [1]. Natural food ingredients with relatively low toxicities commonly consumed in the Asian diet which carries relatively low toxicity have been proposed as one of the best chemopreventive strategies to battle cancer [2]. Fermentation is one of the oldest and most common methods to preserve food in Asia [3]. Fermented foods project a natural and healthy image of Asian diet [3] and are found to possess chemopreventive effects against breast cancer which had been related to the immunostimulatory effects of fermented products [4]. 

Mung bean (*Vigna radiate*), commonly known as “green gram,” is a common source of protein in the Asian diet [5]. A previous study had reported the *in vitro *cytotoxicity and immunomodulatory effects of mung bean sprout thus indicating it as a potential nutraceutical agent against cervical and liver cancers. Mung bean sprout extract had been found to stimulate tumor necrosis factor-alpha (TNF-*α*) and interferon gamma (IFN-*γ*) which consequently stimulated cell-mediated immunity. Furthermore, mung bean sprouts extract also induced cell cycle arrest and apoptosis on the tested cancer cells [6]. Besides germination, products that contained enzyme hydrolysed [7] or fermented [8] mung bean have also been identified as potential cancer chemopreventive or therapeutic agents. We have previously reported that fermentation can significantly improve GABA content of mung bean [9]. GABA has been reported to inhibit cholangiocarcinoma [10] and leukemia cells [11]. To date, the roles of the antioxidant and immunomodulatory effects of GABA enriched fermented mung bean in the prevention of breast cancer are still not fully understood. Thus, this study was aimed at evaluating the antioxidant, chemopreventive, and immunostimulatory effects of GABA enriched fermented mung bean extract on low and high risk 4T1 injected mice. 

## 2. Materials and Methods

### 2.1. Chemicals and Antibodies

Phosphate buffer saline (PBS), Folin-Ciocalteu reagent, hypoxanthine, xanthine oxidase, superoxide dismutase, and tamoxifen (positive control) were purchased from Sigma-Aldrich, USA. Griess reagent was obtained from Invitrogen, USA. Foetal bovine serum (FBS) was purchased from PAA, Austria. Fluorescein (FITC) conjugated anti-mouse CD4 and Phycoerythrin (PE) conjugated anti-mouse CD8 antibodies, mouse interleukin 2 (IL-2), interferon gamma (IFN-*γ*), and tumor necrosis factor (TNF-*α*) ELISA kits were purchased from BioLegend, USA. *Rhizopus* sp.strain *5351 *inoculums were obtained from the culture collection center of the Malaysian Agricultural Research and Development Institute (MARDI).

### 2.2. Cell Preparation

Yac-1 and 4T1 cell lines were purchased from ATCC, USA. Both cell lines were maintained in RPMI-1640 medium with 10% FBS at 37°C, 5% CO_2_. 

### 2.3. Fermentation of Mung Bean

Fermentation of mung bean and the content of GABA were carried out according to our previous report [9]. The fermented mung bean contained 0.122 g/100 g of dried fermented mung bean powder. 

### 2.4. Animals

Female Balb/c mice of 8 weeks old were purchased from the Institute of Bioscience, Universiti Putra Malaysia, and were housed under 12 hours of light and darkness and fed with standard laboratory pellet diet and water *ad libitum*. This study was approved by the Animal Care and Use Committee of Universiti Putra Malaysia. 

### 2.5. In Vivo Breast Cancer Development Experiment

Mice (total of 72 mice, *n* = 8 per group) were separated to 9 groups and pretreated with either PBS (Groups 1–3), tamoxifen (1 mg/kg body weight) (Groups 4 and 5), 200 mg/kg body weight (Groups 6 and 7), or 1000 mg/kg body weight (Groups 8 and 9) of fermented mung bean extract p.o. for 30 days continuously. On day 30 after treatment, 4T1 cells were harvested and inoculated s.c. on the upper portion of the right hind thigh of mouse with either 1 × 10^6^ (high risk for Groups 3, 5, 7, and 9), or 1 × 10^4^ (low risk for Groups 2, 4, 6, and 8) viable cells. Group 1 was injected with PBS and served as normal control. Mice were monitored for 14 days and treatments were continued during this period. Mice were weighted and tumor sizes were measured every 3 days. Tumor volume was calculated according to the following formula: tumor volume = 0.5 × (*W* ^2^ × *L*) (*W* = smaller perpendicular diameter;*L* = larger perpendicular diameter) [12]. On day 21 postinoculation of the 4T1 cells, all mice were anesthetised with 2% isoflurane (Merck) and sacrificed by cervical dislocation. Spleen, tumor, and serum were collected and subjected to the following assays.

#### 2.5.1. Immunophenotyping of Spleen CD4 and CD8 T Cells

Spleen was harvested, washed with PBS 3 times, and pressed through 80 *μ*m sterile wire mesh. The filtrate was then pelleted, treated with lysis buffer (8 g NH_4_Cl, 1 g Na_2_ EDTA, 0.1g KH_2_PO_4_, pH 7.4), and washed with PBS. Then, splenocytes were stained with 10 *μ*g/10 *μ*L of CD4-FITC and CD8-PE antibodies. After that, cells were washed 3 times and resuspended in PBS and subjected to flow cytometry analysis using FACSCalibur flow cytometer (BD, USA). 

#### 2.5.2. Splenocyte Cytotoxicity Assay

Isolated splenocytes (Effector-E) were cocultured with Yac-1 (Target-T) cell in the ratio of E : T at 2 : 1 and 10 : 1. The cytotoxicities of splenocytes from different treatments were determined using the CytoTox 96 nonradioactive cytotoxicity assay kit (Promega, USA) and the percentage of cytotoxicity was calculated according to the user manual [13]. Each sample was assayed in triplicates. 

#### 2.5.3. Serum IL-2 and IFN-*γ* Levels

Collected blood was spun at 8000 g for 5 minutes and subjected to IL-2 and IFN-*γ* determination using the ELISA kit (BioLegend, USA) according to the user's manual [13]. Each sample was assayed for triplicates.

#### 2.5.4. Spleen and Tumor Antioxidant and NO Levels

Harvested spleen and tumor were homogenized in ice-cold PBS by passing through 80 *μ*m sterile wire mesh. All filtrates were pelleted and the supernatants from the spleen and tumor of all groups were subjected to superoxide dismutase (SOD), malondialdehyde (MDA), and nitric oxide (NO) assays [9].

#### 2.5.5. Tumor Histopathology Evaluation

Harvested tumor was fixed (10% formalin), embedded in paraffin, sectioned, stained with haematoxylin and eosin, and viewed using bright-field microscope for histopathological changes according to [9]. 

### 2.6. Statistical Analysis

The results were expressed as mean ± standard deviation (S.D.) and one-way ANOVA followed by Duncan test to analyse the significant level of the treated group compared to the untreated Groups 2 and 3, respectively. 

## 3. Results

### 3.1. Body Weight and Tumor Size Monitoring

As is shown in Table 1, reduction of body weight was correlated well with the increment of tumor size throughout the experiment. Treatments with high concentrations of fermented mung bean and tamoxifen were able to delay the formation of tumor in the low risk group of mice. In the case of the high risk group of mice, these treatments only helped to reduce the size of the tumor throughout the experimental period. Low concentration of fermented mung bean was less effective against reduction of tumor size in the high risk group of tumor.

### 3.2. Spleen CD4 and CD8 T Cell Immunophenotyping

**Figure 1:**
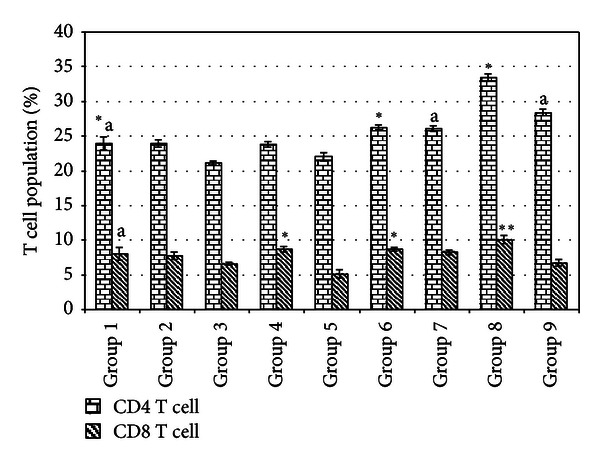
CD4 and CD8 immunophenotyping of spleen from different treatment groups on day 21 after 4T1 cell inoculation. Each value represents the means ± S.D. for three mice in triplicate each. The differences between the control or treated group and untreated group were determined by one-way ANOVA (*for low risk groups while ^a^for high risk groups; *P* ≤ 0.05). Group 1: normal mice; Group 2: untreated 1 × 10^4^ 4T1 cell (low risk) inoculated mice; Group 3: untreated 1 × 10^6^ 4T1 cell (high risk) mice; Group 4: tamoxifen (1 mg/kg b.w.) treated 1 × 10^4^ 4T1 cell (low risk) inoculated mice; Group 5: tamoxifen (1 mg/kg b.w.) treated 1 × 10^6^ 4T1 cell (high risk) inoculated mice; Group 6: fermented mung bean (200 mg/kg b.w.) treated 1 × 10^4^ 4T1 cell (low risk) inoculated mice; Group 7: fermented mung bean (200 mg/kg b.w.) treated 1 × 10^6^ 4T1 cell (high risk) inoculated mice; Group 8: fermented mung bean (1000 mg/kg b.w.) treated 1 × 10^4^ 4T1 cell (low risk) inoculated mice; Group 9: fermented mung bean (1000 mg/kg b.w.) treated 1 × 10^6^ 4T1 cell (high risk) inoculated mice.

Flow cytometry CD4 and CD8 T cell immunophenotyping was carried out to evaluate the influence of fermented mung bean treatment on the change of the T cell population in the spleen. Tamoxifen treated groups showed similar percentages of both CD4 and CD8 T cells as compared to the untreated groups. On the other hand, fermented mung bean was able to increase both the CD4 and the CD8 T cell populations in a dosage-dependent manner (Figure 1).

### 3.3. Spleen Cytotoxicity Assay

**Figure 2:**
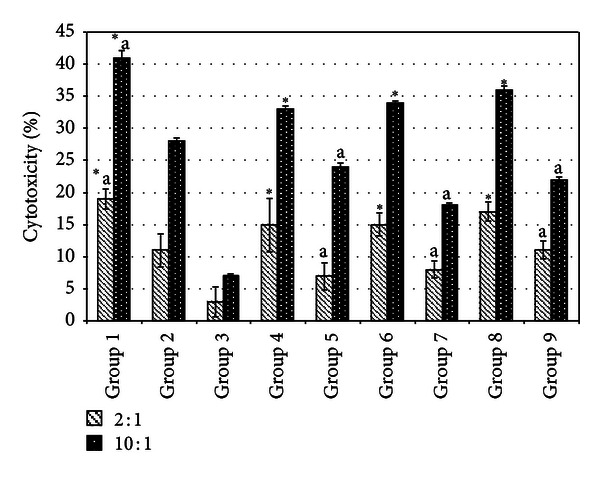
Cytotoxicity level of splenocyte on Yac-1 at E : T ratio of 2 : 1 and 10 : 1 from different treatment groups on day 21 after 4T1 cell inoculation. Each value represents the means ± S.D. for three mice in triplicate each. The differences between the control or treated group and untreated group were determined by one-way ANOVA (*for low risk groups while ^a^for high risk groups; *P* ≤ 0.05). Group 1: normal mice; Group 2: untreated 1 × 10^4^ 4T1 cell (low risk) inoculated mice; Group 3: untreated 1 × 10^6^ 4T1 cell (high risk) mice; Group 4: tamoxifen (1 mg/kg b.w.) treated 1 × 10^4^ 4T1 cell (low risk) inoculated mice; Group 5: Tamoxifen (1 mg/kg b.w.) treated 1 × 10^6^ 4T1 cell (high risk) inoculated mice; Group 6: fermented mung bean (200 mg/kg b.w.) treated 1 × 10^4^ 4T1 cell (low risk) inoculated mice; Group 7: fermented mung bean (200 mg/kg b.w.) treated 1 × 10^6^ 4T1 cell (high risk) inoculated mice; Group 8: fermented mung bean (1000 mg/kg b.w.) treated 1 × 10^4^ 4T1 cell (low risk) inoculated mice; Group 9: fermented mung bean (1000 mg/kg b.w.) treated 1 × 10^6^ 4T1 cell (high risk) inoculated mice.

Cocultivation of splenocyte with Yac-1 cell was used to evaluate the cytotoxicity of the splenocyte on day 21 following inoculation of 4T1 cell. A higher ratio of effector (splenocytes) was associated with greater cytotoxicity against Yac-1 cells. However, this effect was significantly (*P* < 0.05) lower in the untreated mice. Treatments with both tamoxifen and fermented mung bean were able to maintain higher levels of splenocyte cytotoxicity as compared to the untreated group of mice (Figure 2).

### 3.4. Serum IL-2 and IFN-*γ* ELISA Quantification

**Figure 3:**
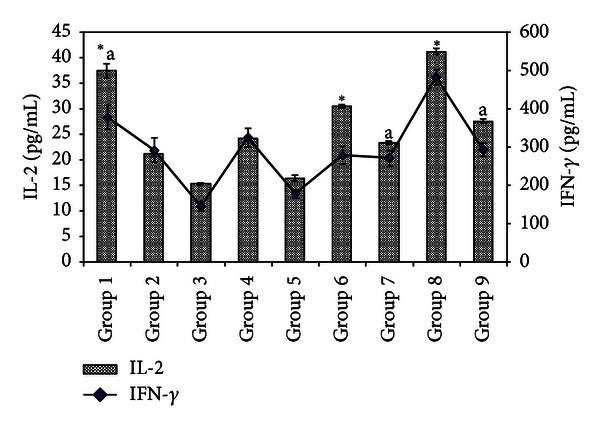
Serum levels of IL-2 and IFN-*γ* (pg/mL) from different treatment groups on day 21 after 4T1 cell inoculation. Each value represents the means ± S.D. for three mice in triplicate each. The differences between the control or treated group and untreated group were determined by one-way ANOVA (*for low risk groups while ^a^for high risk groups; *P* ≤ 0.05). Group 1: normal mice; Group 2: untreated 1 × 10^4^ 4T1 cell (low risk) inoculated mice; Group 3: untreated 1 × 10^6^ 4T1 cell (high risk) mice; Group 4: tamoxifen (1 mg/kg b.w.) treated 1 × 10^4^ 4T1 cell (low risk) inoculated mice; Group 5: Tamoxifen (1 mg/kg b.w.) treated 1 × 10^6^ 4T1 cell (high risk) inoculated mice; Group 6: fermented mung bean (200 mg/kg b.w.) treated 1 × 10^4^ 4T1 cell (low risk) inoculated mice; Group 7: fermented mung bean (200 mg/kg b.w.) treated 1 × 10^6^ 4T1 cell (high risk) inoculated mice; Group 8: fermented mung bean (1000 mg/kg b.w.) treated 1 × 10^4^ 4T1 cell (low risk) inoculated mice; Group 9: fermented mung bean (1000 mg/kg b.w.) treated 1 × 10^6^ 4T1 cell (high risk) inoculated mice.

Mean serum IL-2 and IFN-*γ* levels were reduced in untreated 4T1 inoculated mice especially in the high risk untreated mice (Group 3) (Figure 3). Fermented mung bean was able to restore the production of both IL-2 and IFN-*γ* in a dosage-dependent manner for the low risk group of mice. Fermented mung bean restored less IL-2 and IFN-*γ* production but the levels of these cytokines were still higher than those in the untreated and the tamoxifen treated high risk group of mice.

### 3.5. Spleen and Tumor Antioxidant and NO Determination

**Figure 4:**
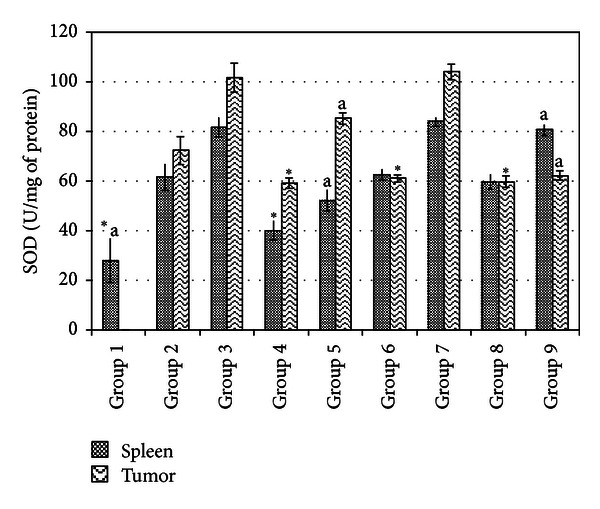
SOD level of tumor and spleen homogenate from different treatment groups on day 21 after 4T1 cell inoculation. Each value represents the means ± S.D. for three mice in triplicate each. The differences between the control or treated group and untreated group were determined by one-way ANOVA (*for low risk groups while ^a^for high risk groups; *P* ≤ 0.05). Group 1: normal mice; Group 2: untreated 1 × 10^4^ 4T1 cell (low risk) inoculated mice; Group 3: untreated 1 × 10^6^ 4T1 cell (high risk) mice; Group 4: tamoxifen (1 mg/kg b.w.) treated 1 × 10^4^ 4T1 cell (low risk) inoculated mice; Group 5: Tamoxifen (1 mg/kg b.w.) treated 1 × 10^6^ 4T1 cell (high risk) inoculated mice; Group 6: fermented mung bean (200 mg/kg b.w.) treated 1 × 10^4^ 4T1 cell (low risk) inoculated mice; Group 7: fermented mung bean (200 mg/kg b.w.) treated 1 × 10^6^ 4T1 cell (high risk) inoculated mice; Group 8: fermented mung bean (1000 mg/kg b.w.) treated 1 × 10^4^ 4T1 cell (low risk) inoculated mice; Group 9: fermented mung bean (1000 mg/kg b.w.) treated 1 × 10^6^ 4T1 cell (high risk) inoculated mice.

**Figure 5:**
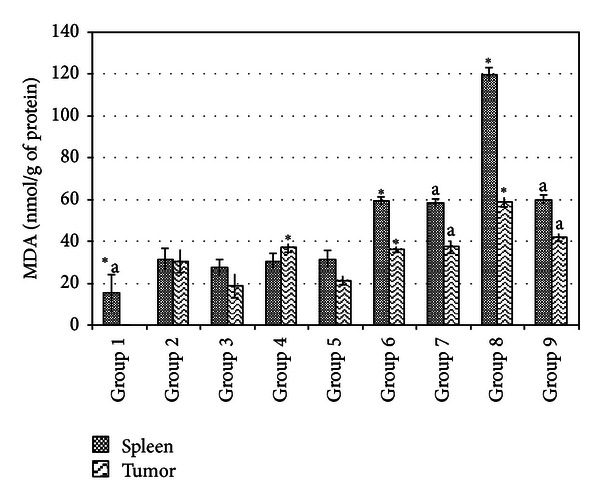
MDA level of tumor and spleen homogenate from different treatment groups on day 21 after 4T1 cell inoculation. Each value represents the means ± S.D. for three mice in triplicate each. The differences between the control or treated group and untreated group were determined by one-way ANOVA (*for low risk groups while ^a^for high risk groups; *P* ≤ 0.05). Group 1: normal mice; Group 2: untreated 1 × 10^4^ 4T1 cell (low risk) inoculated mice; Group 3: untreated 1 × 10^6^ 4T1 cell (high risk) mice; Group 4: tamoxifen (1 mg/kg b.w.) treated 1 × 10^4^ 4T1 cell (low risk) inoculated mice; Group 5: Tamoxifen (1 mg/kg b.w.) treated 1 × 10^6^ 4T1 cell (high risk) inoculated mice; Group 6: fermented mung bean (200 mg/kg b.w.) treated 1 × 10^4^ 4T1 cell (low risk) inoculated mice; Group 7: fermented mung bean (200 mg/kg b.w.) treated 1 × 10^6^ 4T1 cell (high risk) inoculated mice; Group 8: fermented mung bean (1000 mg/kg b.w.) treated 1 × 10^4^ 4T1 cell (low risk) inoculated mice; Group 9: fermented mung bean (1000 mg/kg b.w.) treated 1 × 10^6^ 4T1 cell (high risk) inoculated mice.

**Figure 6:**
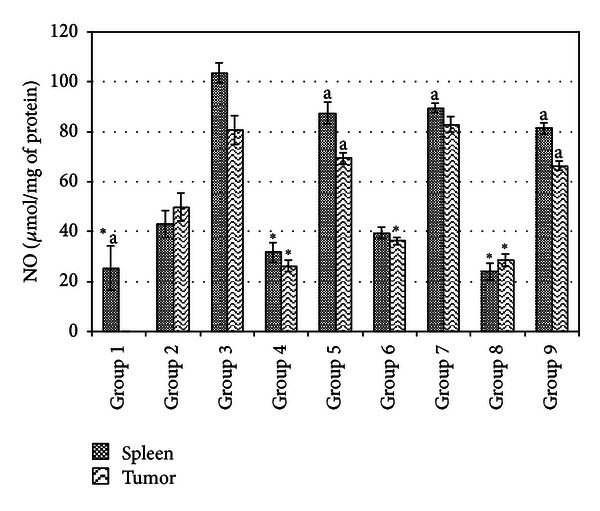
NO level of tumor and spleen homogenate from different treatment groups on day 21 following 4T1 cell inoculation. Each value represents the means ± S.D. for three mice in triplicate each. The differences between the control or treated group and untreated group were determined by one-way ANOVA (*for low risk groups while ^a^for high risk groups; *P* ≤ 0.05). Group 1: normal mice; Group 2: untreated 1 × 10^4^ 4T1 cell (low risk) inoculated mice; Group 3: untreated 1 × 10^6^ 4T1 cell (high risk) mice; Group 4: tamoxifen (1 mg/kg b.w.) treated 1 × 10^4^ 4T1 cell (low risk) inoculated mice; Group 5: Tamoxifen (1 mg/kg b.w.) treated 1 × 10^6^ 4T1 cell (high risk) inoculated mice; Group 6: fermented mung bean (200 mg/kg b.w.) treated 1 × 10^4^ 4T1 cell (low risk) inoculated mice; Group 7: fermented mung bean (200 mg/kg b.w.) treated 1 × 10^6^ 4T1 cell (high risk) inoculated mice; Group 8: fermented mung bean (1000 mg/kg b.w.) treated 1 × 10^4^ 4T1 cell (low risk) inoculated mice; Group 9: fermented mung bean (1000 mg/kg b.w.) treated 1 × 10^6^ 4T1 cell (high risk) inoculated mice.

Lipid peroxidation, antioxidant level, and inflammation of spleen and tumor were measured by MDA, SOD, and NO quantifications, respectively. Figures 4, 5, and 6 show the SOD, MDA, and NO levels of spleen and tumor from different treatment groups on day 21 after inoculation of 4T1 cells. Untreated mice from both low and high risk groups were recorded to have drastic increases of antioxidant enzyme SOD and NO levels indicating that progression of cancer was linked with higher level of antioxidant and inflammation in not only the tumor microenvironment but also in the spleen. On top of this, lipid peroxidation which is indicated by MDA level was drastically reduced. Tamoxifen and fermented mung bean treatments successfully reduced the SOD and NO levels while increasing the lipid peroxidation in both the spleen and tumor of both the low and high risk groups of tumor.

### 3.6. Tumor Histopathology

**Figure 7:**
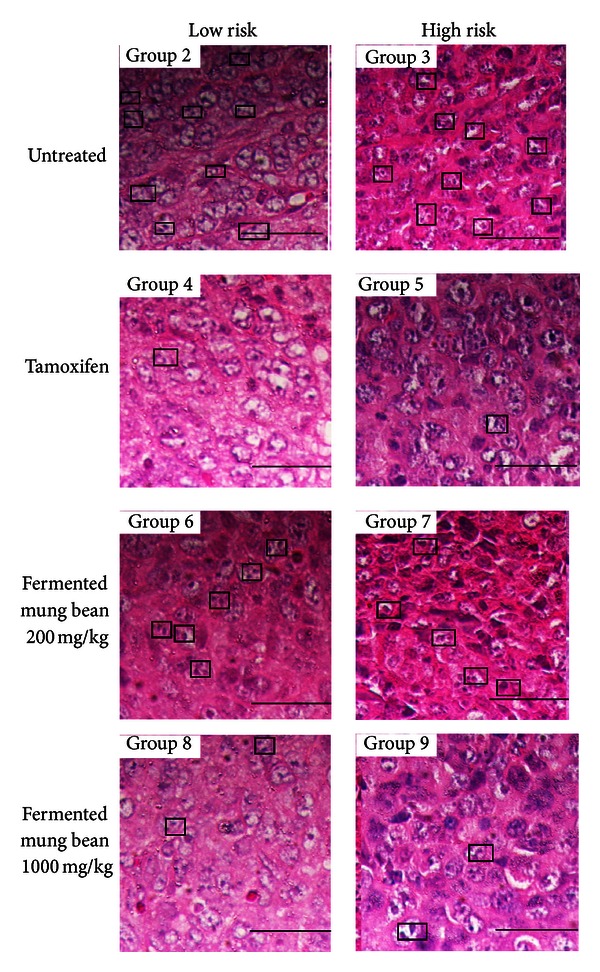
Histological emergence of the 4T1 tumor from Group 2 to Group 9. Boxes indicate cells under mitotic division. Black bars signify 200 *μ*m (magnification 40x). Group 2: untreated 1 × 10^4^ 4T1 cell (low risk) inoculated mice; Group 3: untreated 1 × 10^6^ 4T1 cell (high risk) mice; Group 4: tamoxifen (1 mg/kg b.w.) treated 1 × 10^4^ 4T1 cell (low risk) inoculated mice; Group 5: Tamoxifen (1 mg/kg b.w.) treated 1 × 10^6^ 4T1 cell (high risk) inoculated mice; Group 6: fermented mung bean (200 mg/kg b.w.) treated 1 × 10^4^ 4T1 cell (low risk) inoculated mice; Group 7: fermented mung bean (200 mg/kg b.w.) treated 1 × 10^6^ 4T1 cell (high risk) inoculated mice; Group 8: fermented mung bean (1000 mg/kg b.w.) treated 1 × 10^4^ 4T1 cell (low risk) inoculated mice; Group 9: fermented mung bean (1000 mg/kg b.w.) treated 1 × 10^6^ 4T1 cell (high risk) inoculated mice.

Tumor histology was performed to evaluate the chemopreventive effects of fermented mung bean and tamoxifen. Mitoses were frequently observed in the tumors of the untreated high and low risk groups of mice (Figure 7). Treatments with tamoxifen and high concentration of fermented mung bean were able to reduce mitotic division in the tumors of the low risk group of mice. 

## 4. Discussion

To date, breast cancer still forms the highest percentage among all types of cancer in women [14]. Generally, surgery, radiotherapy, hormonaltherapy, and chemotherapy are the conventional treatments for breast cancer. However, these treatments are always associated with relapse and treatment-induced side effects [15]. Thus, the search for novel agents that can help to reduce the risk of breast cancer incidence is of utmost importance. Chemoprevention denotes the ability to inhibit and reduce tumorigenesis [16]. A previous survey has reported that a high percentage of postmenopausal women with or without history of breast cancer has been utilizing complementary and alternative medicines [15]. In diet based complementary and alternative medicines, many plants that contain various phytochemicals have been identified as good source of chemopreventive agents [16]. Insufficient intake of soy was found to be linked with increased incidences of cancer in developed countries [1] while high intake of legumes including mung bean during adolescence was reported to protect against breast cancer [17]. Mung bean is another choice of legumes that carries multiple health benefits including having antidiabetic, antioxidant, and hepatoprotective effects [9]. Mung bean seeds and sprouts have been recorded to have *in vitro *cytotoxic and immunomodulatory effects and germination was found to significantly improve the bioactivities of mung bean seeds [6, 18]. On the other hand, fermented legume products that contain mung bean as one of the major ingredients have recorded inhibitory effects on the growth of mouse colon cancer xenograft without any side effects [8]. To date, chemopreventive effects on breast cancer solely contributed by fermented mung bean have yet to be reported. Hence, this study was conducted to evaluate the contributions of the antioxidant and the immunostimulation effects of fermented mung beans toward *in vivo *prevention of breast cancer. 

In this study, the transplantable mouse mammary carcinoma 4T1 cells that mimic stage IV breast cancer in humans [15] were inoculated in two different cell densities to resemble high (1 × 10^6^ cells) and low (1 × 10^4^ cells) risk. Based on the measurement of the tumor size after inoculation, low and high concentrations of fermented mung bean were able to delay the formation of breast cancer tumor in the low risk group of mice with efficacies being comparable to tamoxifen. This result was supported by the histopathology evaluation on the tumor where less mitotic division was observed in the fermented mung bean treated group. However, these treatments were less effective in the high risk group of mice. The tumors in both the high and low risk groups were found to have reduced CD4/CD8 T cell populations, low levels of serum cytokines (IL-2 and IFN-*γ*), and impaired spleen cell cytotoxicities. Unlike in the untreated mice, increase of the spleen T cell (both CD4 and CD8) population serum T helper 1 cytokines showed activation of cell-mediated immunity by fermented mung bean that consequently helped to delay the formation of the tumor. IL-2 was able to activate the cytotoxicity and IFN-*γ* production of lymphokine-activated killer (LAK) cell that originated from natural killer (NK) and cytolytic CD8 T (CTL) cells. The synergistic effects of IFN-*γ* and TNF were the major contributors to the stimulation of LAK cytotoxicity [19]. As a result, high levels of IL-2 and IFN-*γ* were found to inhibit tumor formation [4, 13]. The improved cytotoxicity effects of splenocytes isolated from the low risk group of fermented mung bean treated mice against Yac-1 cells may have been contributed by the upregulation of IL-2 and IFN-*γ* production by the extract. Thus, activation of T cells to produce Th 1 cytokines (IL-2 and IFN-*γ*) that further activated cytotoxicity of splenocytes may have contributed to the lower mitotic division in the tumor. 

Overexpression of enzymic and nonenzymic antioxidants was commonly used by malignant cells including breast cancer cells to escape from CTL identification [20]. Moreover, decrease of MDA level was related to severity of breast cancer [21]. Our result was similar with this finding where overstimulation of antioxidant and NO accompanied with decline of MDA level was found in the untreated mice. NO is an important inflammatory mediator that links to the tumorigenesis and angiogenesis of breast cancer *via* the Akt signalling pathway [22]. Many chemopreventive agents including tamoxifen, soy, and green tea were also found to induce anticarcinogenic effects using lipid peroxidation-related pathway besides estrogen and inflammation pathway [21]. For example, isoflavones in fermented soy bean induced breast cancer cell death via promoting generation of ROS which subsequently arrest the cell growth besides carrying antioxidant effect against the normal cell [21]. Thus, increase of lipid peroxidation level by fermented mung bean and tamoxifen treatment in this study may contribute to the reduction of mitotic division in the treated group. In this study, treatment by fermented mung bean and tamoxifen successfully suppressed the overexpression of antioxidant level of spleen and reduced the NO level of the tumor. Reduction of NO level that indicated anti-inflammatory effect has been previously reported as a major event in the chemopreventive effect of resveratrol [23]. Hence, the lipid peroxidation and anti-inflammatory effects of fermented mung bean may indirectly reduce the cancer progression of the 4T1 cells in this study. 

 In this study, fermented mung bean in a dosage-dependent manner has delayed the formation of breast cancer and reduced the mitotic division of the tumor through stimulation of T cell cytokine production (IL-2 and IFN-*γ*) and cytotoxicity. Lower antioxidant and NO levels were observed as compared to the untreated mice from the low risk group. However, fermented mung bean induced poorer chemopreventive effects against mice inoculated with high concentrations of 4T1 cells. Thus, further studies should focus on the effects of long-term consumption of fermented mung beans and evaluation of the detailed mechanism of the chemopreventive action of fermented mung bean against breast cancer. 

## Figures and Tables

**Table 1:** Body weight (g) and tumor volume (mm^3^) changes after inoculation of 4T1 cell up to day 21. Each value represents the means ± S.D. for six mice in triplicate each. The differences between the control or treated group and untreated group were determined by one-way ANOVA.

	Body weight (g)				Tumor volume (mm^3^)		
	Day 0	Day 7	Day 14	Day 21	Day 7	Day 14	Day 21
Group 1	20.12 ± 1.21	22.34 ± 0.68	23.21 ± 0.79^∗a^	25.14 ± 0.79^∗a^	—	—	—
Group 2	21.53 ± 1.08	20.11 ± 0.92	20.14 ± 0.91	18.64 ± 1.33	70.17 ± 6.77	200.33 ± 11.51	400.35 ± 13.41
Group 3	20.75 ± 1.33	19.24 ± 1.13	17.26 ± 1.57	14.32 ± 1.69	300.63 ± 21.32	500.51 ± 21.82	800.68 ± 28.92
Group 4	20.12 ± 0.96	21.32 ± 1.24	21.53 ± 1.82	23.21 ± 1.35*	—	50.12 ± 8.31*	100.62 ± 9.93*
Group 5	20.23 ± 0.77	20.76 ± 0.88	21.43 ± 1.33^a^	21.33 ± 0.91^a^	150.55 ± 9.25^a^	400.38 ± 26.51	540.58 ± 16.79^a^
Group 6	20.18 ± 0.82	21.13 ± 1.23	21.57 ± 0.86	21.57 ± 1.33*	50.42 ± 6.17	100.22 ± 11.51*	270.71 ± 21.36*
Group 7	21.96 ± 1.11	20.11 ± 1.77	18.89 ± 1.59	15.83 ± 1.72	230.36 ± 12.45^a^	400.56 ± 18.35	750.92 ± 31.44
Group 8	20.15 ± 1.12	21.53 ± 0.94	21.16 ± 1.16	22.22 ± 0.94*	—	60.11 ± 8.92*	100.91 ± 11.31*
Group 9	20.84 ± 0.94	20.18 ± 1.26	19.11 ± 1.99	18.55 ± 0.89^a^	170.72 ± 13.55^a^	300.21 ± 21.33^a^	600.39 ± 25.68^a^

Group 1: normal mice; Group 2: untreated 1 × 10^4^ 4T1 cell (low risk) inoculated mice; Group 3: untreated 1 × 10^6^ 4T1 cell (high risk) mice; Group 4: tamoxifen (1 mg/kg b.w.) treated 1 × 10^4^ 4T1 cell (low risk) inoculated mice; Group 5: tamoxifen (1 mg/kg b.w.) treated 1 × 10^6^ 4T1 cell (high risk) inoculated mice; Group 6: fermented mung bean (200 mg/kg b.w.) treated 1 × 10^4^ 4T1 cell (low risk) inoculated mice; Group 7: fermnted mung bean (200 mg/kg b.w.) treated 1 × 10^6^ 4T1 cell (high risk) inoculated mice; Group 8: fermented mung bean (1000 mg/kg b.w.) treated 1 × 10^4^ 4T1 cell (low risk) inoculated mice; Group 9: fermented mung bean (1000 mg/kg b.w.) treated 1 × 10^6^ 4T1 cell (high risk) inoculated mice.

*For low risk groups while ^a^for high risk groups; *P* ≤ 0.05.
